# Shifting From Concept to Practice: The Co-adaptation of Tailored Health Education Training for Truck Drivers

**DOI:** 10.1097/JOM.0000000000003674

**Published:** 2026-02-24

**Authors:** Mohsen Sayyah, James A. King, Vicki Johnson, Louise Hull, Charlotte L. Edwardson, Stacy A. Clemes

**Affiliations:** From the School of Sport, Exercise and Health Sciences, Loughborough University, Loughborough, United Kingdom (M.S., J.A.K., S.A.C.); Leicester Diabetes Centre, Leicester General Hospital, University Hospitals of Leicester NHS Trust, Leicester, United Kingdom (V.J., L.H., C.L.E.); Diabetes Research Centre, College of Life Sciences, University of Leicester, Leicester, United Kingdom (L.H., C.L.E.)

**Keywords:** co-adaptation, occupational health intervention, intervention adaptation, implementation science, lifestyle behavior change, driver health, truck driver

## Abstract

**Objective:**

To co-adapt the SHIFT-UK program into a scalable, accredited, health-focused training module for truck drivers.

**Methods:**

Using a participatory co-creation framework, workshops were held with truck drivers, trainers, and managers. Discussions were transcribed verbatim, pseudo-anonymized, and analyzed using content analysis. Following accreditation and implementation of the co-adapted training module, post-session driver feedback was obtained via an online questionnaire.

**Results:**

The SHIFT-UK program was adapted into an accredited 7-hour driver training module and 1-hour “Short-SHIFT” module component, in partnership with 18 co-adaptors. Short-SHIFT was subsequently delivered to 5500 truck drivers (2023–-2024) within their mandatory training. Feedback from 283 drivers revealed that 77% intended to make healthier lifestyle changes after experiencing Short-SHIFT.

**Conclusions:**

This study demonstrates how participatory co-adaptation can effectively adapt and scale evidence-informed health promotion programs within commercial driver training systems, positively impacting drivers’ health literacy.

## Abstract 2

This study demonstrates how a co-adapted, evidence-informed health education program was successfully translated and implemented for delivery to truck drivers. It highlights a scalable strategy to embed lifestyle health promotion into mandatory training, offering a novel route to improve health outcomes in a high-risk occupational group.

## Graphical abstract

**Figure:**
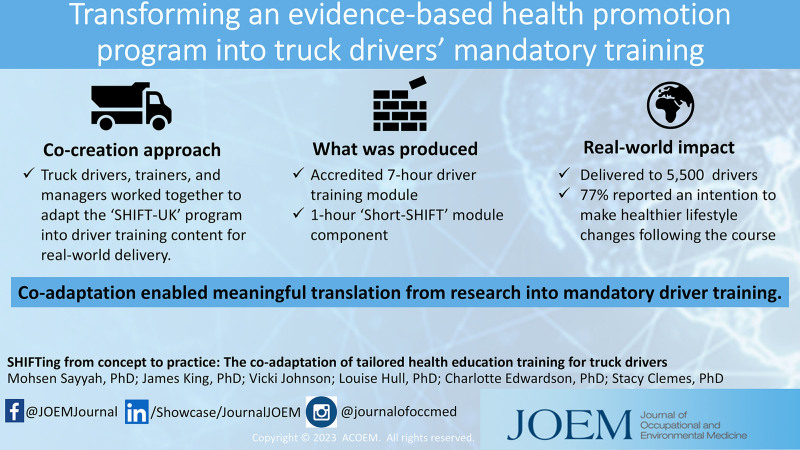

**LEARNING OUTCOMES**

- Understand how the adaptation of a health promotion program from a randomized controlled trial can represent a pivotal step in developing practical training modules for truck drivers.
- Describe the application of a participatory co-creation framework to adapt a health promotion program into a scalable, accredited occupational health training module.
- Recognize how the SHIFT-UK co-adaptation process enhanced the feasibility, reach, and scalability of a public health intervention for a high-risk occupational group.

Truck drivers encounter numerous obstacles associated with their occupation in adopting and/or maintaining a healthy lifestyle. The nature of their work involves extended periods of sedentary behavior, limited opportunities for physical activity, frequent exposure to unhealthy food options, and inadequate sleep due to long working hours and shift work.^1,2^ These challenging working conditions contribute to higher rates of overweight and obesity observed in truck drivers, relative to the general population.^1,3,4^ They are also linked to various obesity-related chronic diseases, such as heart disease, type 2 diabetes, sleep apnea, as well as an increased risk of accidents.^5–9^

Despite the essential role played by truck drivers in global economies, the evidence base for health interventions in this working population is small compared with other occupations.^10^ The absence of health promotion programs for UK truck drivers led to the development of the SHIFT program (Structured Health Intervention for Truckers). Informed by earlier qualitative^11^ and quantitative research,^12^ SHIFT is a theory-driven, multicomponent behavior change program designed to promote positive lifestyle changes in truck drivers. Here, we refer to this program as “SHIFT-UK” to differentiate it from the “Safety & Health Impacts For Truck drivers (SHIFT),” a weight-loss program developed for truckers in the United States.^13^

SHIFT-UK originally consisted of a group-based, interactive 6-hour structured health education session (delivered by 2 trained educators), wearable provision, step count challenges and goal setting, and provision of equipment for a “cab-workout.”^14^ The education session utilized a written curriculum and was designed to support drivers in making sustainable, healthy lifestyle changes, within the constraints of their occupation. The educational component was derived from diabetes self-management and prevention education programs used throughout the UK’s National Health Service^15^ and informed by similar approaches internationally,^16^ tailored for truck drivers. SHIFT-UK was co-created, with industry support, pilot tested,^17^ and refined,^18^ before being formally evaluated within a cluster randomized controlled trial (RCT).^19^ The RCT revealed the program led to a potentially clinically meaningful difference in physical activity and sitting between the SHIFT group and control group (drivers continuing with usual practice).^19^ SHIFT-UK was found to be even more effective in drivers with obesity.^20^ Strong support from drivers and managers was received during the RCT process evaluation for adapting the program into a driver training module, viewed as a route to scale the intervention and provide access to all UK truck drivers.^21^

UK commercial drivers (including truck, bus, and coach) are required by law to complete 35 hours of periodic training, referred to as Certificate of Professional Competence (CPC) training, every 5 years to maintain their licenses.^21^ Driver CPC training is typically delivered in the format of 7-hour taught modules, delivered in-person or virtually. The purpose of the present research was to co-adapt, using an established co-creation framework,^22^ the 6-hour SHIFT structured health education session into a scalable SHIFT-UK driver CPC module. In this context, adaptation is defined as the “thoughtful and deliberate alteration of the design or delivery of an intervention” to improve its fit or effectiveness within a given setting.^23^

## METHODS

### Design

**FIGURE 1.**
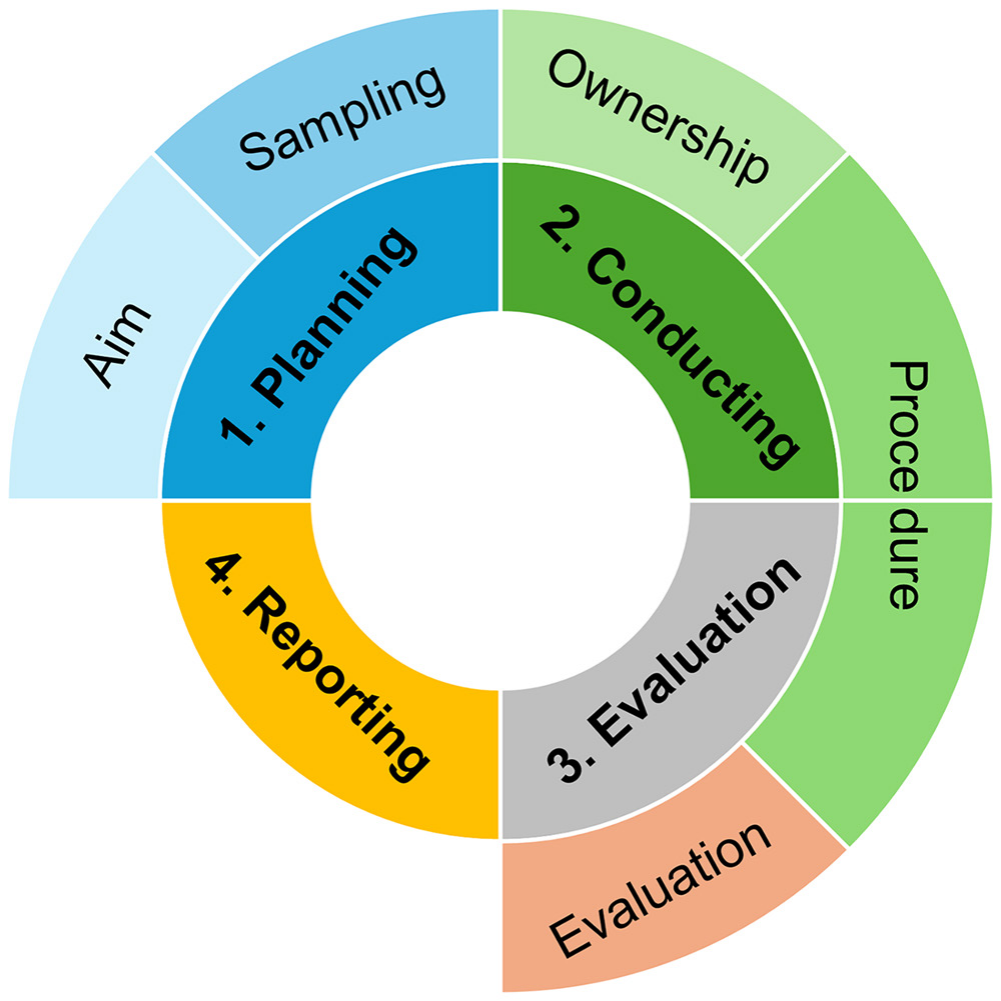
Four stages and five principles adapted from Leask et al.^22^

This mixed-methods study followed the PRODUCES framework (PRoblem, Objective, Design, end-Users, Co-creators, Evaluation, Scalability) proposed by Leask et al.^22^ to guide the systematic co-adaptation of scalable public health interventions for population-level implementation. The 5 key principles of the framework were adopted: (1) framing the aim of the study, (2) sampling, (3) manifesting ownership, (4) defining the procedure, and (5a) evaluation of the co-adaptation process and (5b) evaluation of the co-adapted intervention. In line with Leask and colleagues’ recommendations, the study was structured and reported according to 4 stages: (1) Planning, (2) Conducting, (3) Evaluation, and (4) Reporting (Fig. 1).

To strengthen adaptation transparency, we applied the Framework for Reporting Adaptations and Modifications-Enhanced (FRAME) proposed by Stirman et al.^23^ This enabled classification of planned and unplanned adaptations, the nature and level of modifications, fidelity to core components, and rationale and goals for change. FRAME was used in parallel with the co-adaptation methodology to ensure adaptations were clearly documented and theoretically anchored.

In line with the ADAPT guidance developed by Moore et al.,^24^ which provides a structured, consensus-informed approach for adapting evidence-informed interventions to new delivery systems, populations, or settings, we retrospectively appraised our co-adaptation process against its 4 key steps: (1) assessing the rationale for intervention and intervention-context fit, (2) planning and undertaking adaptations, (3) evaluating adapted interventions, and (4) implementing and maintaining adapted interventions at scale. The work we report here corresponds with Steps 1 and 2. Steps 3 and 4, which relate to formal evaluation and scale-up, are acknowledged as future directions and discussed accordingly. Reporting followed the STROBE guidelines (see Table, Supplemental Digital Content 1, https://links.lww.com/JOM/C387), which presents the STROBE checklist.

### Participants

Eligible participants were recruited from partner logistics companies. The inclusion criteria for co-adaptors were truck drivers (end-users/those expected to benefit from the intervention) and key stakeholders (driver trainers, training and development managers, ie those involved in implementation/delivery of CPC training). A mix of drivers, driver trainers (ie individuals who deliver Driver CPC modules and act as educators), and managers from the transport industry were involved to promote diverse and inclusive participation, fostering a varied comprehension of the topic, and enabling effective discussions among co-adaptors. This approach aimed to stimulate the sharing of experiences and cultivate group interactions and dynamics, and to produce an acceptable and appropriate CPC module suitable for immediate industry adoption and implementation

Ethical approval for the co-adaptation workshops (project ID: 11125) and initial proof-of-concept evaluation (via online questionnaire) (project ID: 16257) was obtained from Loughborough University’s Ethics Review Sub-Committee.

### Stage 1—Planning

As an initial planning activity, we examined in detail the findings from our SHIFT-UK RCT process evaluation,^21^ which included feedback from drivers and transport managers suggesting that the education session could be adapted into a CPC module. Given that all commercial drivers are required to complete CPC training to maintain their licence, this was considered a practical and scalable delivery route for SHIFT-UK and therefore became the focus of our co-adaptation efforts. We then held a SHIFT-UK public engagement/consultation event attended by 32 delegates, including truck drivers, driver union representatives, logistics health and safety directors/managers, training managers, and risk assessors, representatives from professional bodies for individuals working in logistics, supply chain, and transport, representatives from private health companies and consultancies, healthcare professionals involved in program development, and academics. The event included a presentation detailing the creation and evaluation of the SHIFT-UK program, followed by initial suggestions for the translation of the education component into a driver CPC module, based on the RCT process evaluation. Feedback on these initial suggestions was sought during the event, along with further recommendations. The feedback and information gained during this event were used to inform and appropriately frame the co-adaptation workshops, ensuring the workshops had a clear structure and goal.

### Stage 2—Conducting: Co-adaptation Workshops

A comprehensive series of workshops were organized to gather insights for the development of a SHIFT-UK CPC module. To ensure the inclusion of a diverse range of expertise from relevant transport industry stakeholders and drivers, the workshops comprised 2 dynamic in-person sessions and one collaborative online workshop via Microsoft Teams. The workshops were scheduled to last a maximum of 3 hours each, with the in-person and online events following an identical structure. At the outset of each workshop, the research team clarified their roles to the co-adaptors, emphasizing an equal status across all attendees to facilitate shared ownership of the co-adaptation process. Engagement and openness within discussions were encouraged, and attendees were reminded that all discussion points and opinions would remain confidential to the group attending. An informal atmosphere was fostered to stimulate active contributions and diverse expressions of opinions and perspectives. Workshop participants provided written informed consent for their participation before the workshop formally commenced.

A breakdown of each workshop’s content and structure is provided in Table 1. Each workshop commenced with a brief presentation (led by S.A.C.) detailing the background to the SHIFT-UK program and how the program was created and evaluated. This presentation served to clearly identify the occupational health challenges faced by drivers (eg inactivity, poor diet, disrupted sleep), and to upskill co-adaptors on the program content and evidence base. To effectively convey the essence of the program, we streamlined the original 6-hour SHIFT-UK education session into a condensed 1-hour version and integrated this within each workshop (delivered by V.J.). The 1-hour version served as a pivotal component, offering a glimpse into a condensed version of the SHIFT-UK program. The objective was to elicit co-adaptors’ insights regarding content, delivery style, and feasibility of translating it into a comprehensive health-focused CPC module. Following participation in the condensed SHIFT-UK session, the content and allocated time (for each topic area) of the original 6-hour SHIFT-UK education session were shared with co-adaptors. A co-adaptation discussion session followed, facilitated by S.A.C. and V.J. using predefined semi-structured questions, to delve into co-adaptors’ opinions and feedback. In addition to discussing the original 6-hour SHIFT-UK education session, we also sought co-adaptors’ opinions on the feasibility of embedding the other elements of the SHIFT-UK program (wearable provision to self-monitor physical activity, cab-workout, text messages) into a SHIFT-UK CPC module. The discussion sessions were audio recorded, and field notes were compiled (by M.S. and J.A.K.) for an in-depth content analysis.

**TABLE 1.** Outline of the Educational Component of the SHIFT Co-adaptation Workshop

Sections	Content	Duration (min)
1	Welcome and Introduction to the SHIFT project*	10
2	SHIFT RCT findings (upskilling)	10
	One-hour SHIFT introductory session:	
3	Introduction	5
4	Health problems and risk factors	18
5	Physical activity	15
6	Food choices	15
7	Next steps	7
8	Co-adaptation discussion	100

* Transparency with framing the aim and ownership.

### Stage 3—Evaluation

An intended output of the co-adaptation process was the development of a SHIFT-UK driver CPC module for initial implementation by a partner logistics operator, enabling a proof-of-concept delivery and evaluation. In line with the 4-stage participatory methodology developed by Leask et al.,^22^ evaluation planning began early in the process to ensure preparedness for testing following implementation. All UK driver CPC modules must receive accreditation by the Driver and Vehicle Standards Agency^25^ before implementation. This comprised the first step of our evaluation process. Anonymous online feedback questionnaires (accessible via a QR code) were prepared for use during module implementation, for drivers to complete immediately after experiencing the SHIFT-UK CPC session to obtain initial proof-of-concept testing. Feedback questionnaires were designed to capture immediate impressions, intentions based on the acquired knowledge, and the likelihood of making positive lifestyle changes. The questions were adapted from the SHIFT-UK RCT process evaluation feedback questionnaire. For a more in-depth and long-term evaluation, a question was asked about future participation in a follow-up survey to gather feedback on any longer-term behavioral changes. Drivers opting to complete a feedback questionnaire were required to provide informed consent online before completing the questionnaire.

### Stage 4—Reporting

As recommended by Leask et al.,^22^ we completed the checklist for reporting intervention co-adaptation to aid in the transparency of the reporting of this study (see Table, Supplemental Digital Content 2, https://links.lww.com/JOM/C388, which presents the checklist).

### Analysis

The co-adaptation workshop discussions were transcribed verbatim, and pseudo-anonymized transcripts were analyzed using content analysis,^26^ alongside the incorporation of field notes.

**FIGURE 2.**
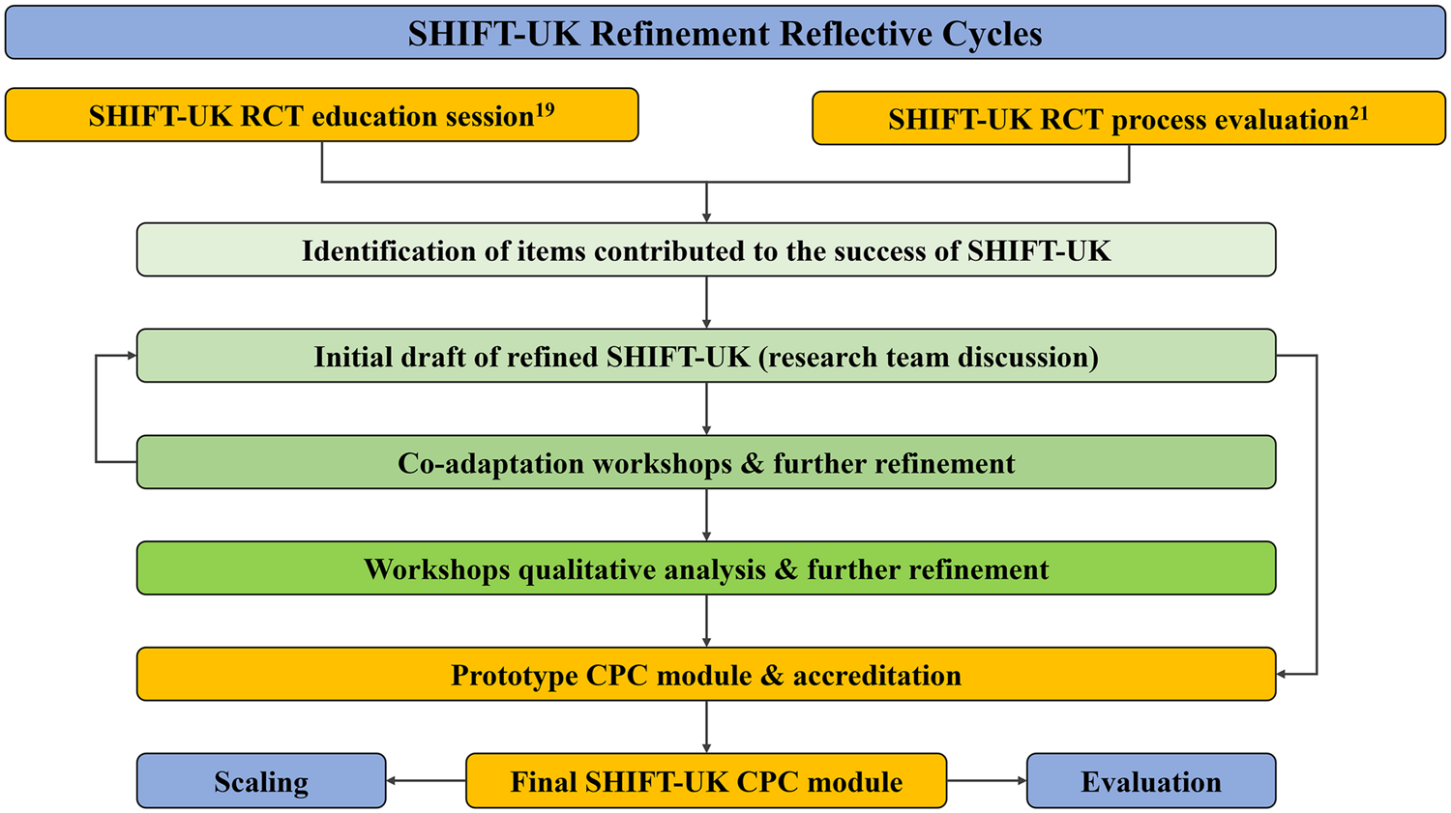
An iterative process of developing SHIFT-UK CPC module.

**FIGURE 3.**
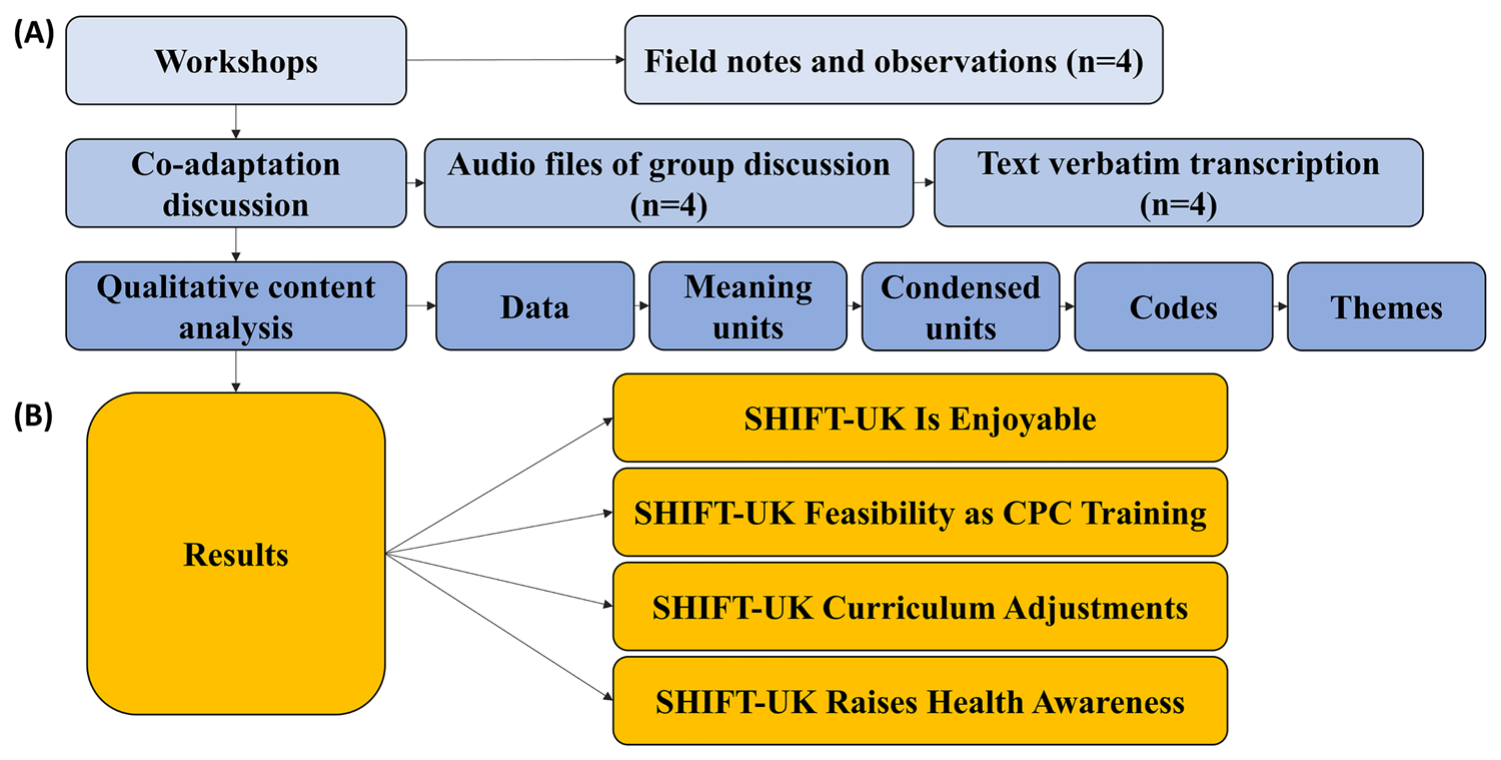
(A) Content analysis methodology flowchart and (B) themes generation process.

Qualitative analyses were led by M.S., who undertook an initial immersion in the comprehensive dataset generated from the co-adaptation workshops. Data were analyzed inductively through an iterative process (Fig. 2). As identical questions were set in all co-adaptation discussion sessions, a summary table for each session was compiled to facilitate the comparison of responses and feedback, and discussed with S.A.C. Subsequently, we focused separately on the manifest content, utilizing the complete discussion session as a unit of analysis. We then compared and reached an agreement on the content (M.S. and S.A.C.). As semi-structured questions were used in each workshop to prompt feedback discussion, responses for each item/question were identified as meaningful units, abstracted into condensed meaningful units, labeled with a code, and subsequently classified as themes (Fig. 3).

Quantitative data derived from the online feedback questionnaires after SHIFT-UK implementation were analyzed using descriptive statistics.

## RESULTS

A total of 18 participants (11% females), including 2 truck drivers, 13 driver trainers, and 3 training and development managers, participated in the 3 co-adaptation workshops. Eleven participants attended an in-person workshop (workshops 1 [n = 3] and 3 [n = 8]), while 7 attended an online workshop (workshop 2). After the condensed 1-hour SHIFT-UK session, co-adaptors were initially asked whether they enjoyed the session, to which 100% responded “yes.” Co- adaptors were also asked whether they felt it would be feasible to implement a 7-hour SHIFT-UK CPC module within their organization, to which 100% also responded “yes.”

The content analysis, as illustrated in Figure 3B, collectively for all workshops, resulted in 4 overarching themes: (1) SHIFT-UK is enjoyable, (2) SHIFT-UK feasibility as CPC training, (3) SHIFT-UK curriculum adjustments, and (4) SHIFT-UK raises health awareness. Each theme is illustrated with quoted examples, demonstrating the analytical progression from meaningful units to themes.

**FIGURE 4.**
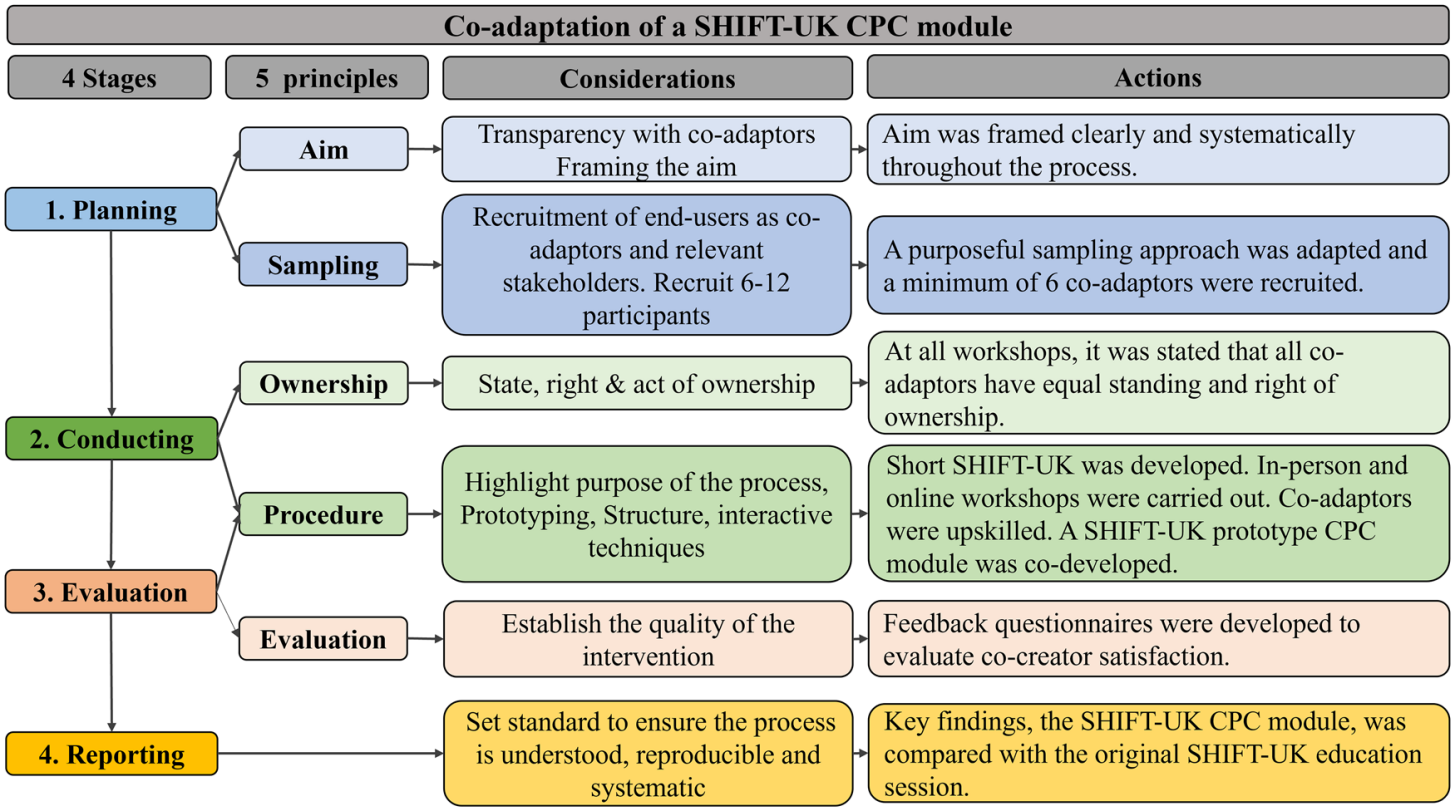
A flowchart of the framework adapted from Leask et al.^22^ for the co-adaptation of the SHIFT CPC module.

Figure 4 presents a flowchart based on the framework adapted from Leask et al.,^22^ illustrating the co-adaptation process of the SHIFT CPC module.

### Thematic Analysis

#### SHIFT-UK Is Enjoyable

The theme “SHIFT-UK is enjoyable” was generated from co-adaptors discussing whether they enjoyed the 1-hour condensed SHIFT-UK session. Overall, all participants agreed that the SHIFT-UK session was enjoyable and different from their previous CPC content.

I wasn’t sure what to expect, but actually it was quite enjoyable, it’s very thought provoking. (Male co-adaptor)

I agree it’s enjoyable. I think it’s very different from our previous CPC that we used to do in sort of 2015, 2017 (in the past). (Male co-adaptor)

In my opinion, it’s a very good one because you’ll get into that health things. It’s very enjoyable. (Male co-adaptor)

#### SHIFT-UK Feasibility as CPC Training

The theme “SHIFT-UK feasibility as CPC training” is about the feasibility of integrating SHIFT-UK into a mandatory driver CPC module, reflecting on the co-adaptors’ opinion on whether the SHIFT-UK program can feasibly be translated into a 7-hour health CPC module for drivers.

Obviously what the business lines choose to do is different argument altogether, it’s feasible to make it (SHIFT-UK) half a day or a full 7-hours CPC module. An hour, yeah, that’s fine, but I think sinking in some people, some drivers, you might need a bit longer. And to get it across, if you put it as an hour, it might work the business line. There’s a lot of transport companies out there, a lot of drivers that have to do this. It’s not option. Make it more usable and more helpful (greater than 3 ½ hour) to the driver community. (Male co-adaptor).

I think it’s (SHIFT-UK) quite important that we put it in. I do honestly, I do think it’s [important] and I think most lorry drivers would tell you the same and I mean everyone also getting bombarded [with information] all this and it’s like what do you do? What do you not do? and that sort of thing is you say it’s thought provoking, but it also makes them realise that… it’s not a massive life change, it’s just little things. And so, to me it’s a really good message that we can across [to] our drivers. (Male co-adaptor)

It’s a brilliant topic. I think it will really fit with our current or next CPC. So, I think this is perfect for this year’s CPC and I think it will fit in very well. (Male co-adaptor)

#### SHIFT-UK Curriculum Adaptation

Table 2 shows the application of the FRAME framework to the theme “SHIFT-UK Curriculum Adjustments,” illustrating how co-adaptors’ inputs were documented using FRAME domains.

**TABLE 2.** Application of the FRAME Framework to the Theme “SHIFT-UK Curriculum Adjustments”

Theme: SHIFT-UK Curriculum Adjustments	Application of FRAME
1. When and how in the implementation process was the modification made?	During co-adaptation workshops, when co-adaptors reviewed the original 6-h SHIFT-UK session.
2. Planned/proactive or unplanned/reactive?	Planned/proactive—adaptations were intentionally introduced during co-adaptation workshops to improve feasibility and engagement.
3. Who determined the modification?	Co-adaptors (drivers, trainers, managers) in collaboration with researchers.
4. What is modified?	Time allocation within the curriculum (ie extending physical and mental health component).
5. At what level of delivery was the modification made?	Group/educational session level (applies to all drivers attending CPC training).
6. Type/nature of modification?	Extension of session time; greater emphasis on health risks and problems (eg depression, sleep, smoking); increased time dedicated to healthier food choices.
7. Fidelity—consistent?	Yes—modifications retained core objectives (raising awareness, supporting healthier behaviors) and did not remove essential program elements.
8. Reasons for the modification (intent/goal + contextual factors)?	(a) Goal: improve feasibility and acceptability by aligning content with drivers’ lived experiences. (b) Contextual factor: co-adaptors reported original time allocations were insufficient for meaningful discussion of key health risks.

#### SHIFT-UK Raises Health Awareness

The theme “SHIFT-UK raises awareness” was generated from co-adaptation workshops where co-adaptors acknowledged the importance of raising awareness of increasing drivers’ physical activity and reducing sitting time, and the uniqueness of the SHIFT-UK program in doing this, and in supporting drivers to facilitate lifestyle changes.

We had something similar to this, but it was more about your sugar intake and your salt intake, and you know, and we add little pictures of some or some little films of exercises and drivers you know sort of doing push ups on next to their lorries and all sorts of stuff you know. But that came across more as telling a driver what to do rather than, I think with the SHIFT program, there’s more just making them aware of what they can do. And then they go away with that option of, well, I’d like to make a change or something like that, you know. (Male co-adaptor)

It’s very interesting. So, it’s not like boy sitting on something that tell him something boring. But it’s yeah, like that you’ve been always, always involved. You’ve been involved. You’re not being spoken to. (Male co-adaptor)

### Seven-hour and 1-hour SHIFT-UK CPC Modules—Co-adaptation

The initial objective of including a 1-hour “taster” of the original SHIFT-UK education session within the co-adaptation workshops was to enable co-adaptors to experience a glimpse into the content and delivery style of the education session, and to facilitate discussions of its adaptation into a CPC module. However, as the co-adaptation workshop discussions unfolded, it became evident that this shortened version held significant potential to be integrated into industry-delivered core CPC modules, which typically focus on road safety and driving-related topics. The co-adaptors felt that the adaptation of a 1-hour version held significant potential for companies, with other core content that needed to be included in their CPC provision, to include an element of health education within their mandatory CPC training. The 1-hour version was termed “Short-SHIFT,” and the final content (Table 3) agreed with co-adaptors. It was agreed with co-adaptors that the purpose of “Short-SHIFT” would be to raise drivers’ awareness about their potential health risks and promote attendance for the full SHIFT-UK CPC module where available, whilst introducing some pragmatic behavioral changes they can implement to predominantly increase their activity and improve their dietary choices.

**TABLE 3.** A Comparison of Time Duration of Each Component of the Content Between the Original 6-Hour SHIFT Education Session, 7-Hour SHIFT CPC, and 1-Hour Short-SHIFT Modules

		SHIFT Programs Duration (min)		
	Content	6 h Original	7 h CPC	1 h Short CPC
A	Welcome and introduction	10	10	5
B	Driver story	30	40	N/A
C	Risks and health problems	45	60	18
	Active break	N/A	10	N/A
	Break	15	15	N/A
D	Depression, sleeping, smoking	30	60	N/A
E	Physical activity	90	90	15
	Break	30	30	N/A
F	Food choices	90	105	15
	Break	N/A	15	N/A
G	Self-management plan	15	30	N/A
H	Next Steps	5	15	7
Total CPC		N/A	420	60
Total including break		360	480	N/A

In addition, co-adaptors considered the feasibility of embedding other elements of the original SHIFT program (wearable provision, cab-workout, text messages). Due to concerns about scalability, logistics, and cost, it was agreed that these components would not be included in the CPC module, with the focus placed solely on adapting the education session for wider delivery.

Adaptations to the original 6-hour SHIFT-UK education session included both planned (eg curriculum extensions), reactive (eg removal of equipment due to scalability constraints), and unplanned (eg development of the 1-hour Short-SHIFT CPC module component) changes. All modifications were co-adapted in collaboration with drivers, trainers, and industry stakeholders and were designed to preserve fidelity to the core objectives and theoretical underpinning of the SHIFT program. A comparison of the time allocated to each component across the original 6-hour SHIFT session, the 7-hour CPC module, and the 1-hour Short-SHIFT is presented in Table 3. To enhance clarity and transparency, all adaptations and their rationale are reported in line with the FRAME framework in Table 4.

**TABLE 4.** Application of the Framework for Reporting Adaptations and Modifications-Expanded (FRAME)^23^ to Adaptations Made During the Co-development of the SHIFT-UK CPC Modules

Educational Component	When	Planned/Unplanned	Who Decided	What Was Modified (Content)	Level of Delivery	Nature of Modification	Fidelity Consistency	Reasons (a) Goal (b) Contextual Factors
Mental health (depression, sleep and smoking)	Co-adaptation	Planned/proactive	Co-adaptors	Drivers’ mental health challenges. Recognizing signs of poor well-being and signposting to support services.	Drivers	Extended time from 30 to 60 min	Fidelity consistent	Goal: not to add new content but to allow richer discussion. Context: mental health needs more time.
Food choices				Drivers’ challenges to adopting healthy dietary behaviors. Challenges of unhealthy, high-fat, high-salt, and high-carbohydrate/sugar-rich roadside food choices.		Extended from 90 to 105 min		Goal: not to add new content but improve fit and engagement. Context: roadside food barriers need more time.
Driver story				Interactive opening activity designed to prompt discussion, reflection, and peer engagement around health risks relevant to commercial drivers		Extended time from 30 to 40 min		Goal: more peer reflection and richer dialog to accommodate larger group in line with DVSA delivery standards. Context: Co-adaptors valued the interactive format and requested additional time to reflect.
Self-management plan				Recognition of different learning styles and preferences among drivers for discussions, questions, and the creation of written action plans.		Extended time from 15 to 30 min		Goal: optional creation of written action plan, enhancing the likelihood of active engagement. Context: accommodate a more detailed explanation of how drivers can utilize different tools effectively and integrate them into their ongoing health management
Next steps	Co-adaptation	Planned/proactive	Co-adaptors	Post-program care pathway and exploration of available support services that drivers could be signposted to. Reflect on the SHIFT-UK CPC module, discuss their experiences, and actively engage in planning their next steps.	Drivers	Extended time from 5 to 15 min	Fidelity Consistent	Goal: enabling a more in-depth exploration of locally available services and resources. Context: drivers wanted pathway/resources.
Activity break				Added activity element		Added a 10-min walk or seated exercise.		Goal: inclusion of active breaks. Context: co-adaptors discussed and supported embedding activity.
Risks and health problems				Extend the time allocated		Extended time for risks and health problems from 45 to 60 min.		Goal: to balance real-life opportunities and constraints, and to facilitate empowerment and self-management, beyond the 7-h session. Context: to plot their results and identify their own health risks after the SHIFT-UK session.
Wearables and cab-workout				Removal of providing drivers with a wearable and cab-workout equipment at scale.	Individual drivers	Removing equipment and replacing it with driver’s own devices and signposting them to online resources (eg Steps4Healths^27^)	Fidelity Consistent: Despite adaptation made, total time for the physical activity section remains at 90 minutes.	Goal: improve feasibility and scalability. Context: cost and logistics challenges.
Text messages	Implementation	Planned/reactive	Researchers	Exclusion of supportive text messages	Individual drivers	^27^Replacing supportive text messages with signposting drivers to freely available digital tools (eg Steps4Health^27^)	Fidelity consistent: digital signposting offers similar features as activity tracking and goal setting	Goal: allowing flexibility to accommodate local or employer-led alternatives to improve feasibility and scalability. Context: scalability, cost, and logistical barriers.
Short-SHIFT	Co-adaptation	Unplanned/reactive	Driver training and development managers	Creation of 1-h “taster” of the SHIFT-UK RCT education session to provide a glimpse into the content and delivery style of the program.	Drivers	1-h version as a standalone module held significant potential to be integrated into industry-delivered core CPC modules.	Fidelity consistent: retains the original goals, maintains theoretical orientation, and acts as a bridge to the full intervention.	Goal: improve scalability and industry alignment. Context: core CPC constraints prevented 7-h integration

### Alignment With the ADAPT Guidance

#### ADAPT Step 1: Assessing Intervention-Context Fit

The co-adaptation process began with a problem-driven approach, identifying key occupational health risks among truck drivers, including physical inactivity, poor diet, limited access to healthy food options, and disrupted sleep due to shift work. These health challenges were assessed in relation to the structure and constraints of mandatory Driver CPC training. Insights from the SHIFT-UK RCT process evaluation, alongside consultations with CPC trainers and industry managers, guided a feasibility assessment. The mismatch between traditional intervention delivery and the realities of CPC training highlighted the need for a tailored education module.

#### ADAPT Step 2: Planning and Undertaking Adaptations

A series of structured in-person and online co-adaptation workshops involving end-users and stakeholders informed the planning and implementation of adaptations. Key modifications included extending sections on the driver story, mental health and food choices, removing wearable technology and health assessments (due to cost and feasibility), and adding an activity break. The Short-SHIFT component also emerged as a pragmatic adaptation that increased scalability while aligning with the program’s delivery style, tone, and theoretical orientation (eg Social Cognitive Theory). However, as it omits some components of the original intervention, such as structural goal setting and action planning, it does not fully preserve all theoretical elements or behavior change techniques. Further evaluation is required to assess its impact and theoretical fidelity (see Table, Supplemental Digital Content 3, https://links.lww.com/JOM/C389, which details mapping of the process to the ADAPT checklist).

Steps 3 and 4 of the ADAPT framework, evaluating the adapted intervention and implementing and maintaining it at scale, were not part of the co-adaptation phase and thus fall outside the scope of this paper. However, a proof-of-concept evaluation was conducted for the Short-SHIFT component, and wider implementation of the 7-hour SHIFT CPC module is planned. These later stages of the ADAPT process will be addressed in future evaluation work and are discussed further in the Discussion.

### Evaluation

#### Accreditation of the SHIFT-UK CPC Module and Module Component

In partnership with a UK-based logistics operator (employing ~8000 truck drivers), who are accredited to develop and deliver driver CPC training in-house, we obtained accreditation by the Driver and Vehicle Standards Agency of the 7-hour SHIFT-UK CPC module and Short-SHIFT CPC module component. One of the accreditation criteria was authorizing expertise in the subject matter for trainers who deliver the module. While a health background is not essential to deliver the SHIFT-UK program, to meet this accreditation criterion, and to ensure the SHIFT-UK program is delivered as intended, driver trainers are required to receive training in SHIFT-UK delivery. Our partner operator was keen to embed Short-SHIFT within their new mandatory 7-hour CPC module for 2023–2024 delivery. To facilitate the implementation of Short-SHIFT into this operator’s mandatory training, their accredited 65-driver trainers were trained to deliver Short-SHIFT during a half-day training event (led by V.J.). Seven driver trainers were subsequently trained, during a 2-day training event, to deliver the 7-hour SHIFT-UK CPC module, which this operator intended to provide as an optional module to their driver workforce (in company time) from 2025 onwards. The proof-of-concept evaluation reported later, therefore, focuses on Short-SHIFT.

#### Short-SHIFT Implementation—Proof-of-concept Evaluation

Between October 2023 and September 2024, approximately 5500 truck drivers, employed by our partner operator, experienced Short-SHIFT as part of their mandatory CPC training. Immediately after the Short-SHIFT session, drivers were invited to complete a nonmandatory, brief, anonymous online feedback questionnaire, accessed via a QR code presented on a slide. As the questionnaire was voluntary and completed in drivers’ own time, the response rate was modest. Feedback questionnaire responses were received from 283 drivers (5% of attendees, sample characteristics: 97% male; mean (±standard deviation) age: 47.4 ± 10.4 years; body mass index 29.7 ± 5.9 kg/m^2^, working duration as a truck driver: 16.4 ± 11.4 years).

Quantitative responses demonstrated that 83% reported finding Short-SHIFT interesting and informative; 78% agreed it raised their awareness of the benefits of physical activity, reducing/breaking up sitting, and a healthy diet. After experiencing Short-SHIFT, 77% reported that they intended to make healthier lifestyle changes, of which the majority (63%) indicated that they planned to modify their dietary choices.

Qualitative feedback captured within free-text boxes on the questionnaire was positive, with drivers particularly commenting favorably on the content and style of delivery. Comments included: *“Best so far – relevant and interactive,” “It’s an eye opener,” “Small lifestyle changes can make a big difference,” and “I enjoyed the course – it was different and informative!”*

## DISCUSSION

This study utilized an established co-creation framework^22^ to adapt a scalable and implementable 7-hour SHIFT-UK driver CPC module, after an earlier evaluation of the SHIFT-UK health promotion program for truck drivers.^19^ While co-adaptors played a pivotal role in this knowledge translation project, careful considerations were made to retain the core elements of the original SHIFT-UK program, preserving the overall content, structure, and delivery style while making necessary adjustments to fit the extended duration required for a comprehensive CPC module. This ensured that alterations did not deviate the SHIFT-UK program from its original format, maintaining coherence and effectiveness in delivering health-promoting practices.

One unexpected (ie, unplanned, in FRAME terminology^23^) but valuable outcome of our co-adaptation workshops was the emergence of the 1-hour Short-SHIFT session. During initial engagement with transport companies, it became clear that companies, bound by mandatory content such as vehicle safety in their annual CPC modules, could not readily incorporate a 7-hour SHIFT-UK program as a core CPC module. Short-SHIFT was therefore designed based on driver trainer feedback and industry needs, with the purpose of embedding into existing industry-delivered CPC modules. Short-SHIFT is not a comprehensive health promotion program but is instead a brief intervention designed to raise drivers’ awareness of looking after their health, and of the importance of physical activity, reducing/breaking up sitting, and a healthy diet. Short-SHIFT was embedded into our partner company’s mandatory CPC module, ensuring their ~8000 truck drivers will experience this training. A further goal of Short-SHIFT in the context of this company was to signpost drivers to the comprehensive, evidence-based, optional 7-hour SHIFT CPC module.

The adaptive evolution of Short-SHIFT showcased its versatility, transitioning from a tool for co-adaptor engagement to a strategic component embedded within the mandatory CPC module for truck drivers. This development represents an innovative solution tailored to both the co-adaptation process and the broader logistics sector’s training requirements. Our initial evaluation of Short-SHIFT, through feedback questionnaires completed by drivers immediately after the Short-SHIFT session, revealed that the session appeared to be effective in raising drivers’ awareness about the importance of adopting healthy lifestyle behaviors. Notably, over three-quarters of drivers reported intending to make healthier changes, a promising response given the session’s brief format and lack of formal goal-setting or action-planning components. A longer-term evaluation, involving the collection of objective physical activity and health data, is needed, however, to fully determine the impact of Short-SHIFT.

We retrospectively applied the FRAME framework,^23^ which provided a structured and transparent method for documenting the adaptations made during the co-adaptation of the SHIFT-UK CPC module. While the Leask et al.^22^ framework guided the participatory co-adaptation process, FRAME allowed us to classify the nature, timing, intent, and fidelity of modifications. This dual-framework approach ensured that the adapted content remained aligned with the original program objectives while accommodating practical implementation constraints such as time limits, scalability, equipment feasibility, and delivery model variations. Importantly, documenting adaptations using FRAME^23^ ensured the structured and comprehensive reporting of adaptations, expanding far beyond simply reporting what was adapted. FRAME also highlighted the delicate balance between maintaining fidelity and enhancing contextual fit, a consideration particularly critical for complex, real-world workplace interventions.

Although we used the original FRAME framework to document adaptations, we also acknowledge the development of the FRAME-IS extension,^28^ which expands the original framework to capture modifications to implementation strategies. The SHIFT-UK adaptation process aligns well with FRAME-IS principles, incorporating multilevel decision-making, both planned and reactive changes, and modifications to content, training, and delivery. Nonetheless, FRAME was retained due to its compatibility with our participatory methodology and the types of content-level adaptations undertaken. Together, Leask’s participatory framework and the FRAME framework offered a methodologically robust and transparent structure for reporting co-adaptation processes, one that could serve as a model for future interventions requiring stakeholder engagement and real-world scalability.

In addition, our work aligns with the ADAPT guidance which^24^ provides a structured, consensus-informed process for adapting evidence-informed interventions to new populations, delivery systems, and settings. By retrospectively mapping our process to the first 2 steps of the ADAPT framework, assessing intervention-context fit and planning/adapting intervention content, we demonstrate that the adaptation of SHIFT-UK into a Driver CPC module followed recognized best practices for contextualization without compromising program integrity.

The alignment with ADAPT principles further reinforces the rigour of our co-adaptation process. We also applied the ADAPT guidance as a reflective checklist to assess whether key adaptation decisions were appropriately grounded in stakeholder input, contextual realities, and implementation feasibility (see Table, Supplemental Digital Content 3, https://links.lww.com/JOM/C389, which illustrates the checklist). While this study applied the first 2 steps of the ADAPT guidance to structure and reflect on the contextualization of SHIFT-UK, we acknowledge that steps 3 (evaluation of the adapted intervention) and 4 (implementation and maintenance at scale) remain ongoing. The Short-SHIFT module component has undergone preliminary proof-of-concept evaluation, but formal long-term assessment of behavioral outcomes, implementation fidelity, and cost-effectiveness has not yet been conducted. These components are essential for understanding sustained impact and will be integral to the future evaluation of the 7-hour SHIFT CPC module. As such, our use of ADAPT represents a phased, real-world application of the framework, consistent with its iterative, context-sensitive approach. Taken together, the use of three distinct but complementary frameworks, Leask’s participatory, FRAME, and the ADAPT guidance, strengthens the transparency, reproducibility, and theoretical robustness of this intervention adaptation case study.

Data collected from the feedback questionnaires will serve as an instrument to gain a holistic understanding of the immediate and longer-term impacts of the SHIFT-UK CPC module and Short-SHIFT module component. As the module continues to be rolled out in our partner company and is now being adapted for implementation within a large bus company, with potential for use in other transport businesses, the evaluation components will offer valuable insights into drivers’ experiences, intentions, and the effectiveness of the SHIFT CPC training in promoting positive health behavior changes. Furthermore, the prospect of utilizing company-level data, such as sickness absence records and accident data, provides a promising avenue for exploring the overall impact of the SHIFT CPC module on drivers’ well-being over a longer-term period.

In conclusion, the successful translation and implementation of the SHIFT-UK program into a real-world setting demonstrates an impactful trajectory in health promotion for UK truck drivers. The SHIFT-UK CPC module and Short-SHIFT have the potential to make a significant contribution to the health and well-being of an at-risk and vital workforce, who have been underserved in terms of health promotion efforts, along with improving road safety for all.

## ACKNOWLEDGMENTS

We gratefully acknowledge the truck drivers, driver trainers, transport managers and our partner operator who participated in the co-adaptation workshops and generously contributed their time, experience, and insights to support the development of the SHIFT-UK CPC module.

## Supplementary Material


