# Supplementary material for: Shifting From Concept to Practice: The Co-adaptation of Tailored Health Education Training for Truck Drivers
Source: J Occup Environ Med. 2026 Feb 24;68(7):e495–505. doi: 10.1097/JOM.0000000000003674 (PMC13378748; doi:10.1097/JOM.0000000000003674)
Supplement: Supplementary file 2 [file joem-68-e495-s002.pdf]

SDC 2: Completed checklist for reporting intervention co-adaptation using the PRODUCES framework for the SHIFT-UK CPC module (adapted from Leask et al., 2019)[22]

	Section	Checklist Item	SHIFT-UK CPC Module
**Planning**	Frame the aim	1. Use each element of the PRODUCES framework (Problem, Objective, Design, end-Users, Co-adaptors, Evaluation and Scalability)	Co-adaptation (Design) a scalable accredited (Scalability) 7-hour health education Programme (Objective) with truck drivers, trainers, and managers (Co-adaptors) to address poor health (PRoblem) and evaluate (Evaluation) in truck drivers (End-users)
	Sampling procedure	2. Inclusion criteria	Recruiting a diverse and inclusive participation of truck drivers, driver trainers, and training managers
		3. In what settings did sampling occur?	Recruitment from a partner logistic company.
		4. How many individuals engaged as co-adaptors (academic / non-academic stakeholders)?	18 total, including truck drivers, driver trainers, training and development managers
		5. Description of the co-adaptors	11% female, average age ~47 years, majority male experienced in the truck driving sector
**Conducting**	Ownership manifestation	6. State right and act of ownership	Equal status was emphasised during workshops. Transparent explanation of roles. Group discussions encouraged shared ownership.
	Procedure Components	7. Level of participation	All co-adaptors asked to engage in each discussion points and provide their input.
		8. Presentation of overall aim	Overall aim introduced at the start of workshops and feedback questionnaire.
		9. Purpose of each meeting	Each workshop began with an explanation of the SHIFT-UK background and the purpose of co-adaptation workshop.

	10. Rules and Responsibilities of Participation	Participants were informed about confidentiality, voluntary contribution, mutual respect, their right of equal status within group and asked to share their insights.
**Procedure Methods**	11. Up-skilling the co-adaptors	Co-adaptors were upskilled on SHIFT-UK RCT findings, health behaviours, and CPC training requirements.
	12. What previous evidence was reviewed, and how?	Reviewed SHIFT-UK RCT results and process evaluation findings. Findings presented during workshops.
	13. If a prototype was developed, describe the prototype and the prototyping process	Initial 6-hour SHIFT-UK education session was used and revised through co-adaptor input. A 7-hour CPC module was co-created based on SHIFT-UK.
	14. Frequency and Duration of Meetings	Three workshops: two in-person, one online. Each lasted ~3 hours. Conducted over a short project period.
	15. Examples of interactive techniques or methods used	Card games (calorie deficit and physical activity scenario-based question), curriculum walkthrough, and reflection on practical delivery.
	16. Examples of fieldwork techniques or methods used	Testing created SHIFT-UK with end-users not involved with the process.
	17. Examples of how iteration occurred during the process	Curriculum was revised after each workshop, including content extensions, delivery changes, and incorporation of co-adaptor suggestions.
**Evaluation** Process	18. Explain how co-adaptor satisfaction and contribution evaluated	Attendance tracked. Feedback collected via post-session questionnaires. 100% found the workshop enjoyable and feasible.
	How are results reported back to stakeholders and the public?	Findings disseminated nationally and internationally via conference presentations, publications, academic and industry events.
**Outcome**	19. Explain how the validity of the outcome and the process were evaluated (e.g. face validation, member checking)	Co-adaptors reviewed the SHIFT-UK CPC content during workshops, provided input on whether the materials made sense, were relevant, and fit with their expectations and experiences, and

	20. Explain plans for formal testing of the effectiveness/scalability of the co-created outcome	SHIFT-UK module delivered as CPC across partner workforce; future evaluation to include long-term behaviour change, potential national and international rollout.
	21) Explain outcome of evaluation (if tested)	Short-SHIFT proof-of-concept tested in 6,500 drivers: 77% planned to make health changes; 83% found it informative. Long-term evaluation planned, including behavioural impact and company-level data.
