# Supplementary material for: Shifting From Concept to Practice: The Co-adaptation of Tailored Health Education Training for Truck Drivers
Source: J Occup Environ Med. 2026 Feb 24;68(7):e495–505. doi: 10.1097/JOM.0000000000003674 (PMC13378748; doi:10.1097/JOM.0000000000003674)
Supplement: Supplementary file 3 [file joem-68-e495-s003.pdf]

SDC 3: ADAPT Checklist for SHIFT-UK CPC Module

Box 4 ADAPT Checklist Question	SHIFT-UK CPC Adaptation Responses
**Have you involved an appropriate range of stakeholders?**	Yes – drivers, trainers, managers, union reps, logistics companies, CPC trainers, and health researchers participated in co-adaptation workshops.
**Is your team clear on roles and decision-making processes?**	Roles were made transparent at workshops; equal status encouraged. Academic team facilitated, but co-adaptors contributed to all content decisions.
**Did you work with original intervention developers?**	Yes – the original SHIFT-UK developers were directly involved in adapting the programme.
**What is the public health problem?**	High levels of physical inactivity, poor diet, sleep disruption, and obesity among HGV drivers.
**Was more than one intervention considered?**	No – SHIFT-UK was selected due to proven efficacy, stakeholder support, and compatibility with CPC delivery structure.
**How robust is the intervention's evidence base?**	High – SHIFT-UK was evaluated via a UK-based cluster RCT with positive outcomes for physical activity and sitting reduction.
**What are the similarities/differences between original and new context?**	Similar: same target population (truck drivers); Different: delivery context (training vs. research); session duration, and trainer delivery model. The Short-SHIFT adaptation aligns with the original programme's tone and theoretical orientation, but omits some behaviour change techniques such as structured goal setting and action planning. This may affect theoretical fidelity and warrants further evaluation.
**Were there any IP/licensing issues?**	No – the SHIFT-UK content was developed in a research context and adapted in collaboration with original creators.
**What adaptations were needed and why?**	Adjusted duration, removed wearables/health checks,
